# The NifTK software platform for image-guided interventions: platform overview and NiftyLink messaging

**DOI:** 10.1007/s11548-014-1124-7

**Published:** 2014-11-20

**Authors:** Matthew J. Clarkson, Gergely Zombori, Steve Thompson, Johannes Totz, Yi Song, Miklos Espak, Stian Johnsen, David Hawkes, Sébastien Ourselin

**Affiliations:** Centre For Medical Image Computing, University College London, Engineering Front Building, Malet Place, London, UK

**Keywords:** Software platform, Image-guided interventions, Augmented reality, Visualisation

## Abstract

**Purpose:**

To perform research in image-guided interventions, researchers need a wide variety of software components, and assembling these components into a flexible and reliable system can be a challenging task. In this paper, the NifTK software platform is presented. A key focus has been high-performance streaming of stereo laparoscopic video data, ultrasound data and tracking data simultaneously.

**Methods:**

A new messaging library called NiftyLink is introduced that uses the OpenIGTLink protocol and provides the user with easy-to-use asynchronous two-way messaging, high reliability and comprehensive error reporting. A small suite of applications called NiftyGuide has been developed, containing lightweight applications for grabbing data, currently from position trackers and ultrasound scanners. These applications use NiftyLink to stream data into NiftyIGI, which is a workstation-based application, built on top of MITK, for visualisation and user interaction. Design decisions, performance characteristics and initial applications are described in detail. NiftyLink was tested for latency when transmitting images, tracking data, and interleaved imaging and tracking data.

**Results:**

NiftyLink can transmit tracking data at 1,024 frames per second (fps) with latency of 0.31 milliseconds, and 512 KB images with latency of 6.06 milliseconds at 32 fps. NiftyIGI was tested, receiving stereo high-definition laparoscopic video at 30 fps, tracking data from 4 rigid bodies at 20–30 fps and ultrasound data at 20 fps with rendering refresh rates between 2 and 20 Hz with no loss of user interaction.

**Conclusion:**

These packages form part of the NifTK platform and have proven to be successful in a variety of image-guided surgery projects. Code and documentation for the NifTK platform are available from http://www.niftk.org. NiftyLink is provided open-source under a BSD license and available from http://github.com/NifTK/NiftyLink. The code for this paper is tagged IJCARS-2014.

## Introduction

Image-guided interventions (IGI) are medical procedures that use computers to provide virtual overlays of image data, in order to guide the surgeon [7]. Medical image computing has become increasingly complex over recent years, spurred on by the advances in imaging technology itself, and the increase in computing power readily available to perform ever more complex tasks within a time frame that fits within a surgeon’s workflow. Underpinning all of these advances is a fundamental need for software platforms and software development processes that can meet these escalating demands. In this paper, the motivation, architecture, design and exemplar applications of the NifTK software platform are described, detailing how NifTK is used for IGI purposes.

### Background

Cleary and Peters [7] provide a review of some of the history of image-guided surgery (IGS) along with the associated technology and clinical applications and use the term image-guided interventions (IGI) to highlight the drive towards minimally invasive techniques. A rapidly progressing area within IGI is the crossover of computer vision techniques to the operating room, and here, Mirota et al. [20] provide a summary of recent research. The need for open-source software has been recently highlighted [13], and medical image computing platforms compared [3, 32]. Of the existing open-source software packages, Cleary [7] mentions three of the major platforms for IGS/IGI as being 3D Slicer [25], the medical imaging interaction toolkit (MITK) [23] and the image-guided surgery toolkit (IGSTK) [6]. In addition, there is the Public software Library for UltraSound imaging research (PLUS) [16] which interfaces with SlicerIGT,^1^ which is an extension of 3D Slicer, and also the Computer-Assisted Medical Intervention Toolkit (CamiTK) [9]. Despite the prevalence of the term “Toolkit”, a necessary distinction must be made between libraries such as IGSTK, the Insight Segmentation and Registration Toolkit (ITK) [12] and the Visualisation Toolkit (VTK) [27] that have no end-user application and are intended to be used by application developers, and platforms such as 3D Slicer, MITK, PLUS, CamiTK and NifTK that are provided with end-user functionality to be used as is, and also provide the capability to be extended through a variety of means. In this regard, MITK provides for a wide variety of different usage patterns and can function both as an end-user application containing out of the box IGI components [10, 19] or as a library for developers [33]. MITK has been specifically designed for extension by third parties by using a highly modular architecture and a range of extension mechanisms [23].

There have also been various efforts to combine software platforms. Lu et al. [17] demonstrated reduced development time by using open-source software compared with writing from scratch. They combined MITK with IGSTK with MITK providing the image processing, and IGSTK providing fiducial location, registration and tracking. The PLUS project is developed in conjunction with the SlicerIGT project, where components are connected via OpenIGTLink [29]. OpenIGTLink is an open-source messaging protocol, designed for medical applications. PLUS provides a variety of hardware interfaces, focussing mainly on ultrasound-based applications, and uses 3D Slicer for visualisation.

Each of the above mentioned IGI platforms, and also medical imaging platforms such as GIMIAS [15] and Med-INRIA [31], has common components such as ITK and VTK. Increasingly, the community is collaborating and sharing code via the Common Toolkit (CTK).^2^ The NifTK project aims to complement existing projects and simply sees each platform as a vehicle to translate new algorithms and research further towards to the clinic. The examples in this paper illustrate some of our recent interests, where a particular focus of development has been that of laparoscopic surgical procedures where Nicolau et al. [22] provides a recent review.

### Motivation

The Centre for Medical Image Computing (CMIC) at University College London (UCL) has a wide-ranging research programme in computation imaging. The high-level, IGI-related requirements for a software platform were:

1. *Algorithm components should be lightweight and have few library dependencies* Our experience is that most medical imaging researchers are not familiar with large-scale software projects. We have previously developed NiftyReg [21], NiftySeg [5], NiftyRec [24] and NiftySim [28]. By being small and easy to build, they have received many downloads.
2. *Make maximum reuse of existing projects; contribute code back to the open-source community* The code reuse policy particularly applied to Graphical User Interface (GUI) development, as these can take a lot of work to do well. Our previous experience had illustrated that while libraries such as ITK, VTK and IGSTK can be combined to create an application, they provide no overall structure for continuously evolving platform development. MITK was chosen as a central component as it provides a wide range of facilities for flexible and modular design. MITK can be used as a library [33], or as an application framework [23] where the design is inspired by the Open Service Gateway initiative (OSGi).^3^ A recent addition has been the addition of micro-services^4^ enabling a service-oriented architecture, which is now used in NifTK, MITK-IGT [10] and MITK-US [19].
3. *Provide the facility to send data between machines over an ethernet network* Various hardware devices only have drivers for one platform (e.g. Microsoft Windows), and we did not want to restrict researchers to working on an unfamiliar platform. OpenIGTLink was chosen as a transmission protocol for data, such as images and tracking data, and would provide interoperability with platforms such as 3D Slicer and PLUS.
4. *Given limited hardware, it will probably be shared across projects* So, software interfaces should be lightweight. The intention of requirement 4 is that device drivers and data-grabbing software should be installed once, and then the machine is ready to be used on multiple projects. The version of this software should change infrequently. Users should not be required to re-compile large software packages, edit many configuration parameters or set up copies of a large single application for each purpose.

Requirements 1 and 2 may seem contradictory. Requirement 1 refers to pure algorithmic, or numerical code. We find that this type of code is normally written by a research scientist, understood by a relatively small number of people and once tested and published, remains relatively unchanged. So, it should be written in as small a library as possible, to be easily redeployed or repackaged. But with regard to user interfaces, middleware or infrastructure code, these require specialised software engineering skill, must be written reliably and robustly, and may need regularly adapting to new applications and interfaces. So, requirement 2 refers to not reinventing the wheel and reusing code where there is a community of developers that have worked hard on providing excellent robust code. The combination of requirements 3 and 4 means that we require small stand-alone programs, primarily to just grab data and stream it via OpenIGTLink to a workstation on a local or remote machine. Requirements 3 and 4 directly led to NiftyGuide as a separate package.

### System overview

The NifTK platform, as deployed for IGI applications, comprises NiftyIGI, NiftyGuide and NiftyLink.

*NiftyLink* is a small messaging library for sending Open-IGTLink messages. NiftyLink provides a simple client and server model with keep-alive pings and detection of dropped connections. Various utility methods make it very easy for 3rd party code to construct and asynchronously send Open-IGTLink messages.

*NiftyGuide* is a suite of as-small-as-possible individual applications, whose sole purpose is to grab data and send via NiftyLink. There currently exist programs for connection to Northern Digital Inc.^5^ (NDI) trackers such as Polaris Vicra, Polaris Spectra and Optotrak Certus and also ultrasound interfaces using the Ultrasonix^6^ Porta and Ulterius APIs (Application Programing Interface).

*NiftyIGI* is the main workstation application and provides a visualisation platform, accepting data from video sources, ultrasound sources, tracking devices and so on.

The use of separate data-grabbing applications provides loose coupling (few inter-dependencies) of software components. For example, this means that NiftyIGI does not care about where tracking data are coming from, as there is no software link. This means the tracking data can be simulated for testing purposes, and different devices can be tested, without recompiling NiftyIGI. Separate data-grabbing components also provide flexible deployment options with different software components running on different machines. In addition, NiftyIGI can integrate data sources that reside locally within the same process space. For example, grabbing stereoscopic high-definition video requires approximately 355 MB/s bandwidth, which is too high for most networks and so was implemented directly within NiftyIGI.

In the literature, other software such as PLUS [16] sends data to remote applications such as 3D Slicer [25] via Open-IGTLink [29]. It is reassuring to see common software patterns evolving independently and standards being more widely adopted. As the NifTK platform benefits from a community of open-source developers, our aim was to ultimately provide code that the research community can benefit from, extend, modify and improve. This means that reuse of existing design patterns, sharing common platform ideas and aiming for interoperability and compatibility are preferred over developing a new architecture for architectures sake. In this paper, we specifically, albeit briefly, address interoperability.

### Contributions of this paper

Within this paper is the first description of the NifTK platform for IGI purposes. This paper contributes the following:

- A new library called NiftyLink is introduced that can be used to transmit OpenIGTLink format messages using TCP/IP over ethernet networks. NiftyLink provides ease of use and reliability for applications using Qt.^7^
- A description of a multi-threaded data source manager, to manage access to different hardware devices, all streaming data to NiftyIGI at different speeds.
- Novel visualisation screens within NiftyIGI for augmenting video data or ultrasound data with rendered objects.

Furthermore, while not novel in itself, the NifTK platform serves as an integration point for a set of common algorithms such as camera calibration [34], distortion correction [4], dense surface reconstruction [30], point-based registration [1] and surface-based registration [2] into an easy-to-use package. In addition, developers can activate any of the available open-source MITK plugins within NiftyIGI, such as those for diffusion imaging [11], segmentation [18], or indeed the MITK Image-Guided Therapy Toolbox [10] and the MITK Ultrasound Toolbox [19]. As NifTK continues to expand, further plugins will be developed and described in future publications. For the purpose of this paper, we focus on the topics listed above.

## Methods

The NifTK architecture for IGI is now described in detail, followed by a performance analysis and examples.

### Introduction to NiftyLink

The purpose of NiftyLink is to enable the user to send and receive messages based on the OpenIGTLink protocol. The reference implementation of OpenIGTLink on GitHub^8^ provides C++ classes to conveniently use a BSD socket. However, there is still work to do to implement OpenIGTLink messaging within an application. The developer has to consider whether to send synchronously or asynchronously, use multi-threading to maintain GUI performance and decide how to handle low-level networking errors and high-level application errors. For this reason, 3D Slicer and PLUS have implemented their own classes to embed the OpenIGTLink reference implementation into their applications. While these classes are open-source, they would have to be extracted class by class in order to embed into another application, and these classes themselves have other library dependencies. Furthermore, various medical imaging projects such as 3D Slicer, PLUS, MITK, NifTK and CTK^9^ all use Qt.^10^ Qt is a widely used library with support for all major OS platforms that provides networking and threading facilities and GUI widgets.

Thus, the motivation for implementing NiftyLink was to create an easy-to-use and lightweight messaging library that integrates well with the rest of the NifTK platform, works reliably across the various platforms that are supported by NifTK and that can be easily adopted by any other C++/Qt/CMake project. The design philosophy of NiftyLink is to provide compatibility with existing projects that use OpenIGTLink such as 3D Slicer and PLUS, to not deviate from the protocol and to be small and self-contained.

#### Features of NiftyLink

The main features of NiftyLink are currently:

- A server that binds to a single port and accepts multiple client connections. Inbound messages are queued in order. Outbound messages are sent to each client.
- A client that connects to the above server.
- Optional keep-alive status messages, and hence detection of dropped connections.
- Client and server are multi-threaded and asynchronous.
- All messages passed are OpenIGTLink messages.
- Helper functions to aid the developer to easily send common messages such as images and tracking data.
- Centralised, thread-safe logging.
- A stand-alone application that serves as a basic message routing client between two server applications.

#### The NiftyLink TCP server and client

Reading and writing to a TCP socket from a client or a server should be a simple and standardised task. However, the developer must decide whether to use synchronous (blocking) functions or asynchronous (non-blocking) functions. In order not to block the main GUI processing, blocking network calls should be processed in a separate thread, and any socket library offering asynchronous services will be delegating to other threads behind the scene. So, both methods require some knowledge of multi-threaded programing. In addition, there are also a wide variety of error conditions that must be coped with, as networks can be unreliable. The medical imaging researcher however should not be concerned with these details, and simply wants to send and receive messages. Qt provides the QTcpServer and QTcpSocket and a variety of documented examples for general-purpose networking. Both the QTcpServer and QTcpSocket classes provide a synchronous and asynchronous API. The Qt documentation recommends the use of the asynchronous methods as networking is inherently asynchronous. So, NiftyLink provides a client and server that are based on these Qt classes, and thus the implementation benefits from all the functionality provided by Qt.

When starting the NiftyLink server, the developer can decide whether to bind to local, IPv4, IPv6 or Any (default is Any) network interface. The NiftyLink server handles network proxies and management of a configurable maximum (default is 30) number of client connections and provides robust error notification via Qt signals. The server can pause and resume accepting client connections. The client simply specifies a host name and port and binds to the server. Both server and client can then send and receive messages. If multiple clients connect to the same server, the messages are queued by the server in the order they are received from each thread, but thread processing order is not guaranteed by the operating system. Both server and client can be configured to send a regular, short message to indicate that the process is still alive. The opposite end of the connection can be configured to warn if no such message is received. This is useful when the sending process is supposed to constantly stream data and enables quick detection of dead connections.

*Implementation details* NiftyLinkTcpServer is a subclass of QTcpServer. Each client connection is handled by a QTcpSocket running inside a separate thread. The main purpose of NiftyLinkTcpServer is to override the incomingConnection() method which is called by QTcpServer when a new connection is established. The overriden incomingConnection() method creates a NiftyLinkTcpWorker class for each client, connects Qt signals and slots and hands the NiftyLinkTcpWorker to a new thread. The remaining methods in the NiftyLink server are concerned with tidying up threads on destruction, connecting signals and slots, passing signals from clients back to the user and forwarding requests to all connected clients.

The NiftyLinkTcpClient class contains a single QTcpSocket to connect to the server. NiftyLinkTcp Client contains a single NiftyLinkTcpWorker and as with the NiftyLinkTcpServer; the remaining methods are concerned with connecting Qt signals and slots and passing signals back to the user.

The NiftyLinkTcpNetworkWorker class is used by both client and server so that message processing functionality is identical at both ends of the client–server connection. A QTcpSocket signals whenever data are ready to be read and the QTcpSocket is queried for how much data are available. Large images may be fragmented over several packets, and Qt knows nothing about the expected size of incoming data. So, care must be taken to read as much data as possible at each invocation and to fully clear the read buffer. If the amount of data available at the socket is less than a header, NiftyLinkTcpNetworkWorker will wait for the next signal. OpenIGTLink headers are fixed size, and the header contains the size of the subsequent message payload. Once enough data have arrived to at least read a header, a new message of the correct type is then created using a message factory, and data read in piece by piece, potentially over several calls, with the message cached between calls. This avoids the likelihood of the socket timing out, or the need for blocking while waiting for data. Sending out data is performed by calling the QTcpSocket::write(..) method, which sends data asynchronously. This means it is always queued behind the scenes and QTcpSocket takes care of actually sending the data. All errors are caught via signals and passed back to the client or server.

#### NiftyLink message processing

NiftyLink also provides the facility for keep-alive messages. The TCP protocol itself provides keep-alive options, but these simply instruct the kernel to be more proactive in checking the status of a connection, so that when it is next used, the kernel can report quicker whether or not the connection is dead. However, for IGI applications, it may be critical to know when a device that should continuously stream data has stopped sending data. When there are no real data to send, NiftyLink provides a method to send a status message every 500 ms. The other end of the connection can be configured (on/off) to check every 1 s whether any data (data messages or keep-alive messages) have been received. In addition, following [29], it is useful to measure latency from the time a message is created, and starts to be sent, to the time it is fully deserialised and available at the other end. NiftyLink provides functionality to count message statistics during continuous operation and can be remotely triggered to output them as a Qt signal and a message to the log. Finally, NiftyLink provides convenience functions to create common messages such as tracking data and image data messages from a QImage.

*Implementation details* The keep-alive functionality is implemented using an igtl::StatusMessage, deliberately sending STATUS_OK to be compatible with 3D Slicer and PLUS. It is activated by calling SetKeepAliveOn (bool) and optionally SetCheckForNoIncoming Data(bool). The client provides the RequestStats() method which will send an igtl::StringMessage to the remote server containing the string STATS, causing the server to output statistics. Both client and server provide the OutputStats() slot to enable the user to output statistics at their end of a connection.

#### The NiftyLink application

In addition to unit tests, and several testing applications, a small Qt-based GUI was developed that creates two client sockets and reads incoming messages from one socket and writes them to the other socket. This was developed because both PLUS and NiftyIGI open server ports, and this small application could serve as a bridge. Basic filtering was implemented for test purposes to only pass messages of a certain type.

#### NiftyLink logging

NiftyLink offers a centralised logging mechanism based on the QsLog library.^11^ QsLog supports logging to file or console from multiple threads and is essential to track the output from many simultaneously running threads. Log events are automatically tagged by a timestamp and priority (Trace, Debug, Info, Warn, Error, Fatal), and the output messages can then be filtered by priority.

#### NiftyLink changes to OpenIGTLink

The development of NiftyLink resulted in various fixes and features for OpenIGTLink. They have been raised as issues 44, 45, 46, 47, 48, 49, 50 and 56 for the OpenIGTLink project on GitHub,^12^ and available on branches prefixed with the issue number on the NifTK/OpenIGTLink fork.^13^ These issues included minor bugfixes (44, 49, 50) and feature requests to add a message factory (56), transmit error measures with tracking data (47) and setting and retrieving the timestamp in nanoseconds (48). We also used the GetSystemTimeAsFileTime() method instead of clock() on Windows along with NtQueryTimer Resolution and NtSetTimerResolution API to set the timer resolution to 0.5 milliseconds on application start (45). Finally, we also provided error handling fixes to the socket classes (46). By community consensus, 44, 48, 49, 50 and 56 were merged back to OpenIGTLink, and 45, 46 and 47 left on the NifTK fork, as they are not fundamental to the core API.

### Overview of NiftyGuide

**Fig. 1:**
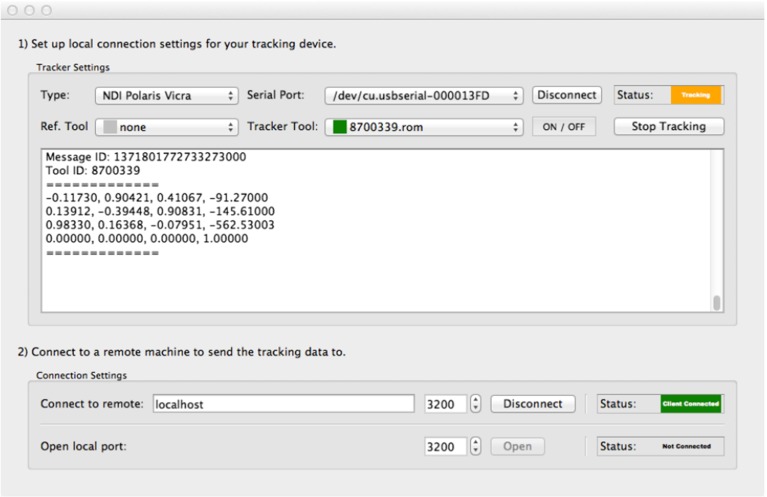
A typical NiftyGuide application: niftkNDIPolaris enables tracking using the NDI Polaris Spectra and Vicra

NiftyGuide is a suite of applications, with each individual application responsible for a single task. For example, NiftyGuide contains a program called niftkUltrasonix to grab images using the Ultrasonix Porta API and another program called niftkNDIPolaris to grab tracking information from NDI Polaris and Vicra optical trackers. The original design philosophy was that each application should have as few library dependencies as possible, and each program does one simple job well. The NiftyGuide package is configured using CMake,^14^ and at build time tries to minimise the number of libraries compiled. Depending on the selected CMake options, these libraries may include IGSTK, and hence ITK and VTK, which will give access to other tracker types via IGSTK for future use. Figure 1 shows a typical user interface. The creation of command line applications for specific tasks is also straightforward.

### NiftyIGI: application level architecture

The main workstation application NiftyIGI uses the MITK application framework. MITK provides an extensible architecture, where each item including the main application window is provided by a plugin, and each plugin is implemented using CTK. The interested reader should refer to the literature to understand the original library design [33], use of dynamically loaded user interface components [18] and a summary of infrastructure facilities provided by MITK [23] that are extensively used by the NiftyIGI application.

#### NiftyIGI additional plugins

The NiftyIGI program provides a number of additional plugins for IGI purposes. The functionality presented here can be summarised as:

1. *Data sources* Provides dynamic management of input data sources such as trackers or imaging devices, enabling logging of data and timestamps to files, playback of logged data and placing the incoming data into the central MITK Data Storage.
2. *Overlay display* Will display the image using a VTK foreground renderer with a fixed camera, and in addition render the 3D scene overlaid on top of the image, thereby providing augmented reality displays, given a 2D image in Data Storage. Provision is made for a calibrated camera model [4, 34] for video images. Alternatively, the camera can be kept at a fixed position relative to the 2D image as the image is moved in space, which is suitable for applications such as freehand ultrasound.
3. *Tracked pointer* Takes tracking data, a calibration file and a surface mesh representation of a pointer, and transforms the surface mesh according to the tracking transformation to display the pointer in 3D space. The pointer tip position is calculated and displayed. MITK Point Sets can be saved a point at a time into Data Storage, and the main display focus point can optionally be updated.
4. *Tracked image* Takes tracking data, a calibration file and an image and transforms the geometry of the image by the tracking transformation, so that the image can be visualised in 3D space. This can be used to display for example an ultrasound image moving in 3D space.

These plugins can be used for a wide variety of uses, see “Image guidance using a Tracked Pointer”, “Image guidance using a tracked video source” and “Image guidance using a tracked ultrasound probe” sections for illustrative examples. The design philosophy of NiftyIGI is that the Data Sources plugin is responsible for inserting data into the Data Storage. All other plugins can be added to provide additional algorithms to process the available data from the Data Storage, ensuring loose coupling of plugins. The visualisation is simply an observation of the current data, using either the available MITK viewers or custom viewers like the Overlay Display. The Tracked Pointer and Tracked Image plugins are relatively simple, simply moving objects in space. We now discuss the remaining plugins in more detail.

#### NiftyIGI: the data sources plugin

A key use-case and system requirement for data input within NiftyIGI is to handle data from multiple sources at different frame rates. An ultrasound machine may send messages at 40 fps and a tracking device at 20–100 fps. This data must all be captured and optionally logged to disk. The architecture is based around a single management class containing a list of sources, with each source having an independent GUI if required to preview the incoming data. These preview GUIs are only created on demand using the ITK ObjectFactory mechanism and was inspired by the creation of segmentation tool GUIs in [18]. In addition to images, point sets, surfaces and meshes being loaded into Data Storage, we also specifically store tracking transformation matrices. This means that *all* incoming data from the QmitkIGIData-SourceManager get pushed to Data Storage, so that plugins *only* have to look in Data Storage, and are unaware of where the data came from. The ability to visualise the position and orientation of a coordinate system helps debugging, and the rendered glyph can easily be made invisible if not needed.

**Fig. 2:**
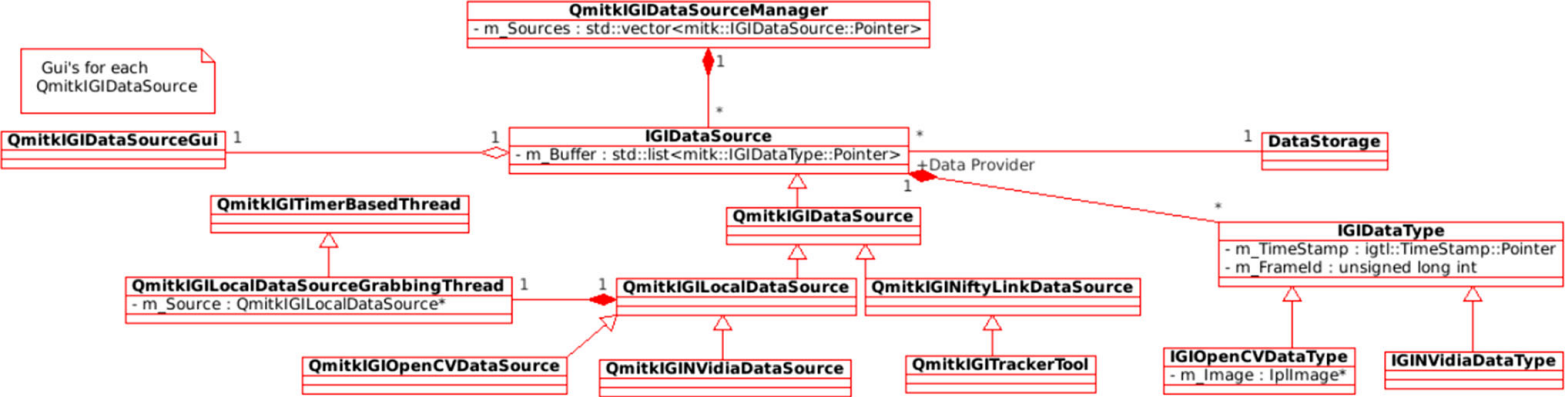
The NiftyIGI Data Sources Plugin: each IGIDataSource manages a buffer of IGIDataType is controlled by QmitkIGIDataSourceGui and contains its own thread to collect data, ensuring GUI responsiveness

*Implementation details* In Fig. 2, the Data Source plugin contains a QmitkIGIDataSourceManager, which can have a dynamically constructed list of IGIDataSource’s. Each IGIDataSource sub-type can define its own implementation for retrieving data, such as from a network socket as in QmitkIGINiftyLinkDataSource, or from a frame-grabber as in QmitkIGIOpenCVDataSource and QmitkIGINVidiaDataSource. Each IGIData Source subclass runs a separate thread to collect the data. A separate clear-down thread, owned by QmitkIGIData Source-Manager will erase these buffers at a configurable frequency. Independently, the QmitkIGIDataSource Manager will use a timer to trigger an update of Data Storage for a given timestamp. Each IGIDataSource then retrieves the closest match from its buffer, calculating the time difference (lag). Once all IGIDataSource’s have been copied to Data Storage, a single event is emitted so that other plugins can synchronise to this update rate, or choose to ignore it, and update at a plugin-specific frequency. The QmitkIGIData-SourceManager timer can be dynamically adjusted using user-specific preferences. Finally, the mitk::Rendering-Manager is asked to update all the viewers. This design enables different frame rates from each source, the calculation of lag and whether each source is up to date. Colour coded warning icons, each colour set by a user-defined preference, indicate when sources are out of date.

#### NiftyIGI: the overlay display plugin

The Overlay Display is a simple customised view component to display a 3D rendered scene merged with an image. There are two distinct modes. “tracked camera” is suitable for display using a moving calibrated perspective camera model (e.g. video camera), whereas “tracked image” places the virtual rendering camera perpendicular to a moving image (e.g. ultrasound image). An additional optional 3D render window can be displayed side by side within the same screen. The image data used are selected from any 2D image in Data Storage and so could be from a live video or ultrasound feed, or individual frames loaded as static images, which is useful while testing, debugging and working without live video sources. NifTK additionally provides command line applications for camera calibration, hand-eye calibration and a plugin for distortion correction.

*Implementation details* The *tracked camera* mode is achieved through a VTK foreground renderer, where the image rendered is the maximum possible size to fit the display window and centred. If the selected image has associated camera intrinsic parameters, the overlay view is scaled according to the intrinsic parameters by virtue of a customised subclass of vtkCamera, which passes the intrinsic parameters to OpenGL directly. Furthermore, the view automatically adjusts to screen resize events, maintaining the calibrated camera view at any screen size. The user can select a tracking transformation from Data Storage whereby the virtual camera position and orientation is continuously updated to provide a view of the 3D rendered scene as if from the viewpoint of the tracked camera device. See Fig. 7.

The *tracked image* mode is achieved by creating a plane within the 3D rendered scene that represents the position, size and orientation of the 2D image. Using MITK mappers, this plane samples a slice of all volume data within Data Storage and uses texture mapping and opacity blending to map image data to the plane. This provides the originally selected 2D image, but also a merged sub-sampling of any 3D volumes such as Magnetic Resonance (MR) or Computed Tomography (CT). The standard vtkCamera is fixed perpendicular to the image plane and made to maximally fill the available window. This plane is a member of the 3D scene; so, all remaining 3D geometry such as surfaces and points are rendered around it. The plane position and orientation is updated via the Tracked Image Plugin, and the vtkCamera follows the movement of the image plane. See Fig. 8.

## Experiments and results

### NiftyLink performance analysis

A series of experiments were conducted to evaluate the performance of NiftyLink and compare it with the original OpenIGTLink implementation on GitHub.^15^ The objective was to assess the frame rate and latency of data transfer for representative examples of tracking and imaging data. Typical requirements within our lab are tracking up to 4 objects, at 30–60 fps. Tokuda quotes tracking requirements of 40–375 Hz, and robotics applications requiring 1 KHz, and tests up to 1,024 fps with 16 matrices per message [29]. Imaging requirements in our lab for example might be ultrasound images at $640 \times 480$ pixels in greyscale 8bit format, at 20 fps, equivalent to 6,000 KB/s, and here, Tokuda targets 4,096 KB/s[29]. Two computers were used:

1. Apple MacBook Pro (mid 2010); CPU: Intel Core i7 dual core @2.66 GHz; memory: 4 GB; OS: OS X v.10.8.5
2. Apple MacBook Pro (late 2013); CPU: Intel Core i7 quad core @2.3 GHz; memory: 16 GB; OS: OS X v10.9.4

These were connected to the college network that provides a gigabit Ethernet link. Clocks were synchronised using the open-source implementation^16^ of the precision time protocol [8] as in [29]. Latency experiments similar to those in [29] were performed for both Open-IGTLink and NiftyLink with computer 2 as sender and computer 1 as receiver. All time measurements were taken using gettimeofday() function defined in 4.3 BSD UNIX and a part of Standard POSIX 1003.1-2001.

#### Latency of tracking data transfer

The first experiment was to measure the performance of transmitting tracking data messages. Latency was defined as the time between the start of generating tracking data at the sender host and the end of deserialisation at the receiver host. The time of the start of serialisation was stored in the timestamp field of the OpenIGTLink message and transferred to the receiver host, where it was compared with the time point to finish deserialisation. The number of channels was 16, meaning 16 tracking matrices per message. 10,000 messages, each containing randomly selected tracking matrices were transmitted at a rate of 128, 512 and 1,024 fps. The results are in Table 1.

**Table 1:** Mean, standard deviation (SD) and maximum (Max) latency of tracking data transfer for OpenIGTLink and NiftyLink software

Frame rate (fps)	OpenIGTLink			NiftyLink		
	Mean (ms)	SD (ms)	Max (ms)	Mean (ms)	SD (ms)	Max (ms)
128	0.30	0.11	0.65	0.34	0.10	1.17
512	0.34	0.11	0.62	0.35	0.10	0.66
1,024	0.31	0.13	0.69	0.31	0.14	0.75

Measurements taken over 10,000 frames of 16 channel tracking data messages

#### Latency of imaging data transfer

The second experiment was to measure the performance of transmitting image data messages. Images were loaded and stored in memory. Latency was defined as the time between the start of copying the image into the OpenIGTLink message and the end of deserialisation of the message at the receiver host. The frame rate was fixed at 32 fps, and the image size varied as 128, 256, 512 and 2,048 KB. 100 image messages were transmitted. In addition, both the OpenIGTLink and NiftyLink client and server were tested streaming $640 \times 480$ greyscale, B-mode ultrasound images at 100 fps, with a mean latency of 3.5 and 3.6 ms, respectively. This requires approx 30 MB/s, which is lower than the capability of the underlying network. The results are in Table 2.

**Table 2:** Mean, SD and maximum latency of image data transfer for OpenIGTLink and NiftyLink software

Image size (KB)	OpenIGTLink			NiftyLink		
	Mean (ms)	SD (ms)	Max (ms)	Mean (ms)	SD (ms)	Max (ms)
128	1.53	0.10	2.37	1.63	0.14	2.74
256	3.05	0.16	4.27	3.12	0.24	5.31
512	5.91	0.20	7.41	6.06	0.33	9.20
2,048	21.27	1.84	25.17	23.53	0.60	29.31

Measurements taken over 100 images, sent at 32 fps

#### Latency of tracking and imaging data

The third experiment was to measure the performance of sending interleaved tracking and imaging messages. The imaging frame rate was varied by $2^n$ fps, with image size of $4{,}096/2^n$ KB/s respectively, with $n$ in $1, 4, 5$, and measurements taken over 100 samples. Tracking data were fixed at 100 fps and 16 channels. The results are in Tables 3 and 4.

**Table 3:** Latencies of tracking data transfers during simultaneous transmission of image and tracking data over the same connection

Frame rate (fps)/image size (KB)	OpenIGTLink			NiftyLink		
	Mean (ms)	SD (ms)	Max (ms)	Mean (ms)	SD (ms)	Max (ms)
2/2,048	0.41	0.69	4.95	0.70	1.92	18.56
16/256	0.34	0.13	0.70	0.39	0.12	0.89
32/128	0.40	0.11	0.60	0.41	0.11	0.64

Tracking data were fixed at 16 channel, 100 fps. The image data transfer was fixed at 4,096 KB/s, and consisted of 100 image messages

**Table 4:** Latencies of image data transfers during simultaneous transmission of image and tracking data in the same experiment as Table 3

Frame rate (fps)/Image size (KB)	OpenIGTLink			NiftyLink		
	Mean (ms)	SD (ms)	Max (ms)	Mean (ms)	SD (ms)	Max (ms)
2/2,048	23.22	0.32	25.78	23.67	0.68	27.98
16/256	2.99	0.15	4.21	3.12	0.22	5.16
32/128	1.50	0.13	2.71	1.68	0.14	2.69

### NiftyIGI performance analysis

The architecture described in “NiftyIGI: the Data Sources plugin” section enables a number of data sources to collect data into independent buffers. At a regular clock tick, the system time is taken, and each buffer in turn asked to update the central Data Storage repository as of the current system time. Once this is complete, all displays are re-rendered. This fourth experiment aimed to evaluate the performance of the fetching and re-rendering process.

A Dell Precision T7600, running Windows 7 Professional, with 2 Intel Xeon E5-2609 2.40 GHz processors, 16 GB memory, NVidia^17^ Quadro K5000 graphics card, and NVidia SDI input card, was used as the main host. A Viking 3D stereo laparoscope^18^ was attached. NiftyGuide was used to capture data from an NDI Polaris Spectra,^19^ an Ultrasonix MDP^20^ and an Ascension 3DG.^21^ NiftyLink was used to stream data into NiftyIGI.

**Fig. 3:**
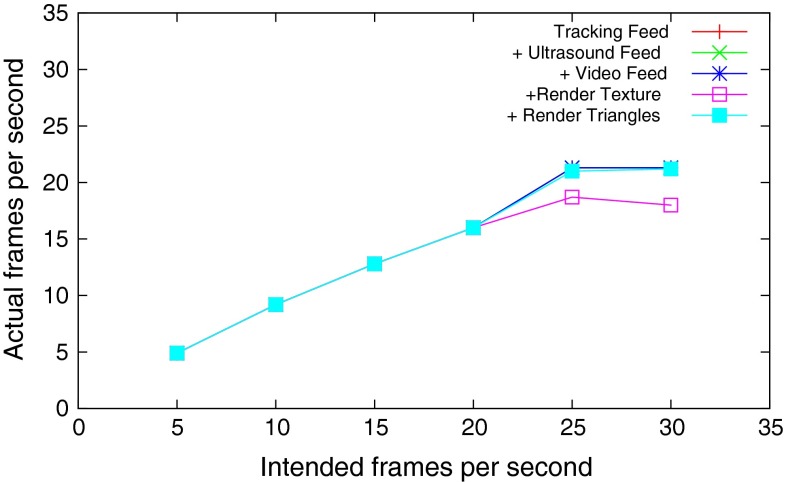
NiftyIGI was increasingly loaded with data feeds, and more rendering tasks. NiftyIGI was set to update at a certain frequency, denoted by “Intended frames per second”. The *actual* frame rate was measured by NiftyIGI. See “NiftyIGI performance analysis” section

**Fig. 4:**
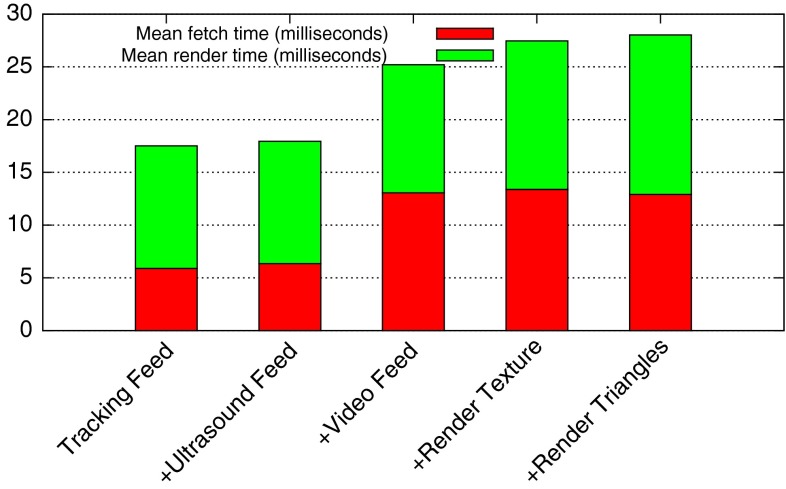
Following from Fig. 3, performance depends on fetching data to update the central Data Storage, and then re-rendering. Fetch and render times were recorded while running NiftyIGI at 5–30 fps for 30 s each and averaged. See “NiftyIGI performance analysis” section

The system was progressively loaded: First, the tracking feed was added, then the ultrasound feed and then the laparoscopic video feed. At this point, there was little to render, just tracking markers/icons. Then, a typical liver surgery set of triangle meshes was loaded containing 863,000 triangles, and containing meshes of the liver, bones, spleen, gallbladder, vasculature and so on. Segmentation and meshing were performed by visible patient.^22^ Then in addition, a texture-mapped plane showing live ultrasound data was added to the 3D scene. At each increase in load, the frame rate was varied from 5 to 30 fps and the time to fetch data from buffers into the central Data Storage and the time to render was measured over continuous operation of 30 s and averaged. Results can be seen in Figs. 3 and 4.

### Use-cases

The examples described below have been performed on various combinations of Windows 7, Scientific Linux 6 and Mac OS X 10.8/10.9 operating systems, using four trackers and two imaging devices.

#### Image guidance using a tracked pointer

**Fig. 5:**
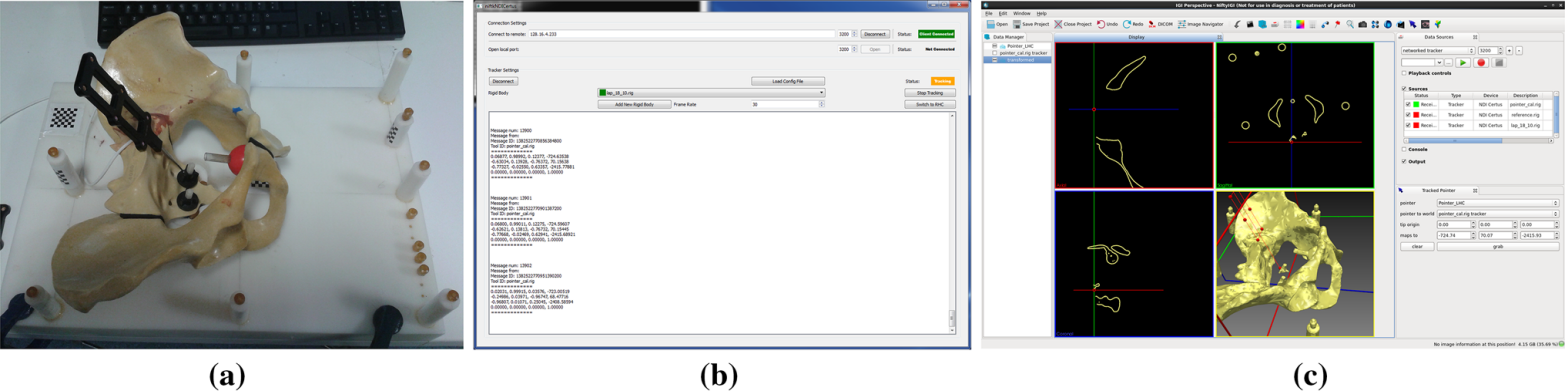
An example of a basic Tracked Pointer system using NifTK. **a** The NDI active pointer is placed within a plastic pelvis phantom (physical layout). **b** niftkNDICertus from the NiftyGuide suite of applications sends tracking data to **c** NiftyIGI which displays 2D outlines and 3D views via the standard MITK Display. The NifTK Tracked Pointer plugin moves the *red* wireframe pointer representation as tracking updates, visualised in the bottom right-hand quadrant of NiftyIGI

**Fig. 6:**
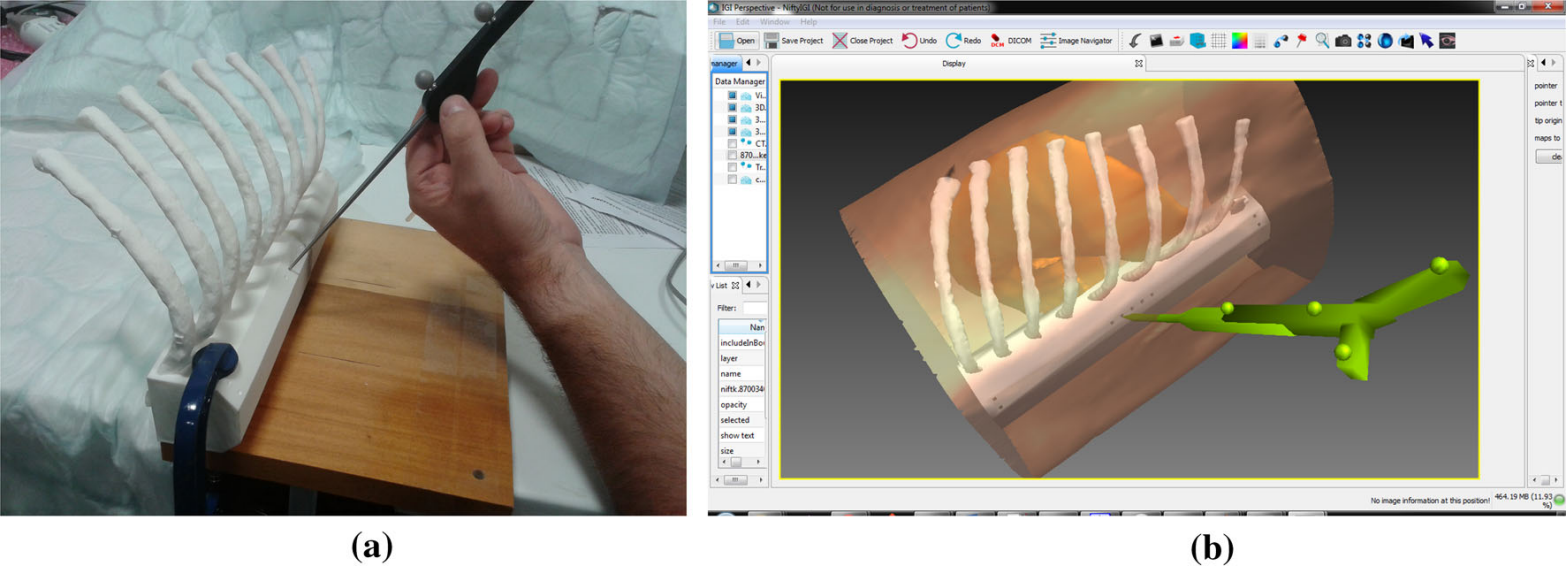
A further Tracked Pointer example, utilising passive tracking in NifTK. A CT scan from a porcine experiment was used to 3D print a ribcage phantom. **a** The physical layout, where tracking is performed using an NDI Polaris Vicra, sampled using niftkNDIPolaris from the NiftyGuide suite of applications. **b** The visualisation in NiftyIGI, using the standard MITK Display, showing only the 3D window

A basic use of image guidance technology is to display the location of a tracked physical pointer, along with a registered pre-operative model. NiftyIGI was set up as follows: An NDI Optotrak Certus was used to track a single NDI 6-IRED active pointer, calibrated via the NDI 6-Degree Architect software. A custom made VTK surface representing the 6 IREDs, and pointer tip was used for visualisation. A plastic pelvis phantom and fiducial markers were attached to a solid acetal base. The phantom and attached fiducial markers were CT scanned resulting in an image that was cropped to $373 \times 137 \times 776$ voxels with $0.914 \times 0.9144 \times 0.5$ millimetres voxel spacing. An iso-surface was extracted using the VTK 5.10 marching cubes implementation. CT points for 6 fiducials were saved as an mitk::PointSet file using the MITK PointSet Interaction Plugin. Corresponding points for the physical location were saved using the NiftyIGI Tracked Pointer Plugin. The mitk::PointSets were registered [1] and applied to the CT surface model. The NiftyIGI Tracked Pointer Plugin was used to update the location of the pointer model and visualise the pointer as it moved. Hence, a basic IGS system is realised, see Fig. 5. A further example is shown in Fig. 6, using an NDI Polaris Vicra tracker and a porcine ribcage phantom.

#### Image guidance using a tracked video source

**Fig. 7:**
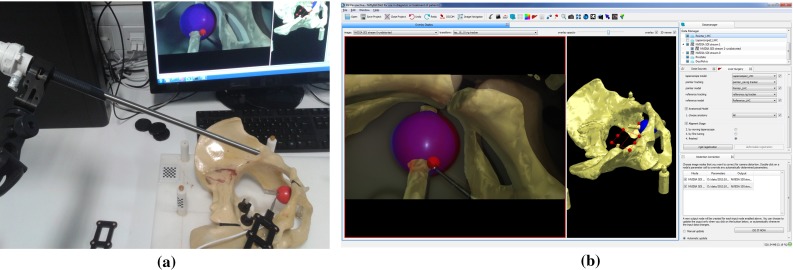
An example demonstrating the *tracked camera* mode in the NifTK Overlay Display. A Viking 3DHD laparoscope was tracked using an NDI Optotrak Certus. The NiftyIGI Overlay Display Plugin enables a calibrated video view to augment video data with rendered 3D data. **a** The physical, tracked laparoscope. **b** The augmented display showing a *blue* representation of the prostate overlaid on the *red* physical prostate giving a purple appearance, and also the Tracked Pointer in *red*

In minimally invasive surgery, laparoscopic or endoscopic video sources provide real-time views of the surgical scene. The NiftyIGI Data Sources Plugin provides a stereo high-definition interface to the NVidia SDI pipeline. Given the correct position, orientation and perspective calibration, image guidance can be achieved by augmenting the video data with virtual rendered objects. NiftyIGI was setup as follows: An NDI Optotrak Certus was used to track the position and orientation of a Viking 3DHD laparoscope.^23^ Both video cameras and the hand-eye calibration were performed using NifTK utilities based on commonly available methods [4, 34]. The NiftyIGI Overlay Display was used in *tracked camera* mode to update the position of a virtual camera as the laparoscope moved. The registration of CT to world coordinates was as in “Image guidance using a tracked pointer” section. Figure 7 shows an example display showing the wireframe mesh of the phantom prostate overlaid on the video, and additionally a rendering of the tracked NDI active pointer. At this magnification, even small errors can be visually apparent.

#### Image guidance using a tracked ultrasound probe

**Fig. 8:**
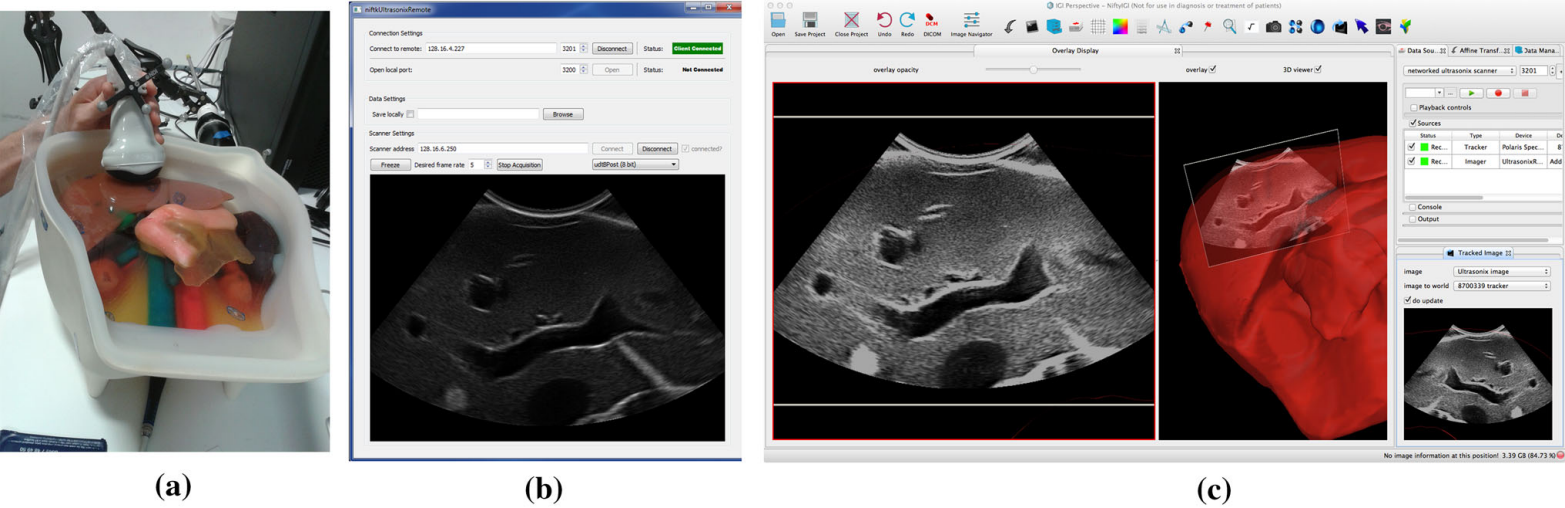
An example demonstrating the *tracked image* mode in the NifTK Overlay Display. Tracking data are provided by an NDI Polaris Spectra, sampled using niftkNDIPolaris from the NiftyGuide suite of applications. **a** Shows the physical setup. In **b** niftkUltrasonixRemote connects via the Ulterius API to an UltrasonixMDP and sends imaging data to NiftyIGI. **c** Shows the NiftyIGI Overlay Display Plugin positioning the virtual VTK camera perpendicular to the Tracked Image (**c**, *left*). Additionally, we can see the tracked ultrasound image moving in the 3D window, intersecting the 3D geometry of the phantom (**c**, *middle*). The transformations are updating using the NifTK Tracked Image Plugin (**c**, *right*)

A third image guidance scenario is to display a virtual representation of an image from, for example, a tracked ultrasound probe, mixed with data from pre-operative scans such as MR or CT. NiftyIGI was set up as follows: An NDI Polaris Spectra was used to track an Ultrasonix 4DC7-3/40 probe, connected to an Ultrasonix MDP scanner. A Kyoto IOUSFAN^24^ abdominal intraoperative and laparoscopic ultrasound phantom was imaged, and live data sent to NiftyIGI using NiftyLink. The NiftyIGI Overlay Display was used to visualise the CT and ultrasound data. Figure 8a shows the physical set-up. Figure 8b shows niftkUltrasonixRemote running on Windows, which connects to the Ultrasonix MDP via TCP/IP using the Ulterius API and then sends imaging data to NiftyIGI running on a MacBook Pro. Figure 8 (c, left) shows the NiftyIGI Overlay Display with the virtual camera fixed perpendicular to the ultrasound image plane. Figure 8 (c, middle) shows a 3D view showing the location of the ultrasound image plane relative to the abdomen phantom components and (c, right), an MITK 2D view showing contours as the liver data intersects with the ultrasound plane.

## Discussion

Performance testing has been performed for the new NiftyLink library and the NifTK platform as a whole. We now discuss each component in turn.

### NiftyLink

The experiments in “NiftyLink performance analysis” section and results in Tables 1, 2, 3 and 4 show that NiftyLink is fractionally slower than OpenIGTLink, but not significantly so. The only noteworthy difference occurs in Table 3, row 1. When sending data, the OpenIGTLink socket class calls the BSD socket send() 25 function which blocks until all data are written to the kernel send buffer. The NiftyLink version however will call QTcpSocket::write(), which queues the data and returns immediately. Thus, in the case of NiftyLink, several subsequent tracking messages will have an earlier timestamp, resulting in an increased measure of latency, even though the arrival time will be comparable. So, while there is a measurable cost to using Qt signals and slots, the difference in performance is negligable.

NiftyLink has proven to be successful for transmission of tracking data and images between computers at a frame rate that is suitable for our applications. Time synchronisation has not yet proven to be a problem in practice as we normally test on various platforms, but deploy to the operating theatre on a single machine. The experiments described in “NiftyLink performance analysis” section were performed on Mac OS X in order to use the open-source ptpd daemon. No such implementation exists for Windows. On Windows, it would be possible to run an NTP server^26^ and synchronise other machines to it.

The use of OpenIGTLink or NiftyLink, which both transmit data over TCP/IP, has implications for safety and could lead to adverse events. For commercial product development, a detailed risk analysis is required to demonstrate compliance with ISO-14971, and a suitable software architecture must be designed. Software must additionally be implemented according to ISO-62304. Very little research work on safety-critical software has been published for IGI systems in general, with the exception being in safety of medical robotics [14]. It would make a significant difference to the timing of a system if images arrive via one mechanism, and tracking data via another. For a research platform, flexibility and the ability to reconfigure the system are key, and a detailed investigation of suitable software architectures for safety critical IGI systems is planned for future work.

### NiftyGuide

Currently, NiftyGuide only contains programs for collecting data from the NDI Polaris Vicra and Spectra, the NDI Certus, Ascension 3DG and an Ultrasonix MDP, which are the hardware currently in use in our laboratory. However, the lightweight design means that it is easy for additional programs to be added, without the need to understand the full NifTK platform. Initial experience suggests that for some developers, it has been easier to write these small programs using the direct API calls provided by manufacturers than using larger software packages such as IGSTK.

The NiftyGuide components simply grab data and send via NiftyLink. Other software packages such as PLUS provide a similar approach [16]. The advantage of using Open-IGTLink is that it provides a mechanism for interoperability. Thus, we do not see NiftyGuide as a competitor to PLUS. Indeed, PLUS provides additional interfaces to devices such as video hardware that can then be used to stream data into NiftyIGI. As time progresses, there will be new hardware, and obsolete hardware, and either NiftyGuide or PLUS could be added to or extended as the need arises.

### NiftyIGI

In this paper, we have described our data management (“NiftyIGI: the Data Sources plugin” section) and visualisation functionality (“NiftyIGI: the Overlay Display plugin” section). The experiments in “NiftyIGI performance analysis” section test the performance of the data management framework with simultaneous use of trackers, video data and ultrasound data, as is typical for an IGS application. In Fig. 3, we can see a measure of performance. The GUI is set to refresh at a certain rate via a QTimer object. In practice, when processing takes too long, either in the timer thread, or with various other threads consuming CPU resources, Qt will opt to miss a timer tick if it can’t keep up with the timing schedule. So,“actual frames per second” are expected to be less than the “intended frames per second”. Note also that in Fig. 3, tasks are incrementally added. When the system is not rendering ultrasound data texture mapped on a plane, the system copes well up until a requested frame rate of 20 fps, or an actual frame rate of 15 fps. When ultrasound data are visualised in the surgical scene, displayed on a texture-mapped plane, as in Fig. 8d (middle), the system drops to about 5 fps. This slow down is not caused by the proposed framework and is due to expensive, repeated updates to OpenGL pixel buffers and is a known performance bottleneck that is being addressed. Figure 4 shows the timing, measured in milliseconds. It can be seen that updating Data Storage with the most up to date data from all sources is of the order of 5–13 milliseconds, whereas rendering could be of the order of 30–65 milliseconds.

The success of the Data Sources plugin stems from the single purpose of just putting data into Data Storage. The proposed architecture enables all sources to operate at different speeds and yet enables the GUI refresh rate to be changed dynamically. Typically we run the refresh rate at 5–15 fps. Our design also permits the simple implementation of a logging and playback mechanism. As each frame of data is processed, it can be saved to disk. Subsequently, the only requirement is that each data source can read files from disk rather than a live source and place the data back into Data Storage. The rest of the application remains the same, as the remaining application logic is unaware of where the data came from.

Our original requirements in “Motivation” section mentioned that algorithm components should be lightweight. The NiftyIGI GUI requires many software libraries and cannot be considered lightweight (see “Appendix”) in the same sense that NiftyReg [21] and NiftySeg [5] are lightweight. However, perhaps, it is more important to consider ways to manage the inherent complexity, and here, it is the MITK application framework that gives us the ability to modularise, encapsulate and isolate pieces of functionality, thereby organising the code well. In comparison with a previous unpublished user interface, the use of the application framework provided by MITK has resulted in more loosely coupled modules, each with clearer purpose, and hence there is less of a tendency for the code base to degenerate. The NifTK build process provides the ability to build multiple user interfaces, each with different combinations of plugins. This has proven useful as the end-users have very different use-cases and do not want unnecessary clutter within their user interface.

As software evolves, newer design styles come to light. Recent work has demonstrated the use of micro-services^27^ within the MITK-US module [19], and a similar approach is now implemented within the MITK-IGT framework.^28^ The micro-services approach is complimentary to the proposed architecture, and future work could investigate utilising the micro-services approach within the NiftyIGI Data Sources manager, and also embedding NiftyLink/OpenIGTLink functionality within MITK itself.

### Interoperability

In addition to the experiments described in “Experiments and results” section, NiftyGuide applications have been tested successfully sending imaging and tracking data into 3D Slicer via NiftyLink. 3D Slicer provides the facility to open a server port and listen for connections or to open a client port, and bind to an already running server. PLUS provides a server program that can broadcast image and tracking data, and client applications that connect to the server. Work is underway to enable NiftyIGI to work more closely with PLUS. The common medium however is OpenIGTLink, and here, NifTK aims to provide compatibility and interoperability rather than directly compete with platforms such as PLUS and 3D Slicer.

## Conclusion

In this paper, the NifTK platform has been introduced within the context of IGI. The motivation, design and initial use-cases have been described and demonstrated. The aim is to provide novel plugins for the various user interfaces and new command line programs, and integrate new research algorithms. The platform benefits from the extensive use of open-source software and CMIC is committed to feeding back code where feasibly possible.

CMIC has previously released smaller sub-components of the NifTK platform such as NiftyReg [21], NiftySeg [5], NiftySim [28] and NiftyRec [24]. These packages are lightweight and research focussed and have been widely adapted due to their simplicity and ease of deployment. In addition, CMIC has contributed towards projects such as MITK and CTK.^29^ This paper describes a much larger suite of end-user applications, incorporating much more complex architectures and library dependencies. It is envisioned that components from this programme of work will be open-sourced as they mature and develop, either by directly releasing the code, or via integration with collaborative projects.

## Appendix

### Library dependencies

Table 5 lists the main libraries used within NifTK, and Table 6 shows the dependencies for each sub-project. Care has been taken to choose unrestrictive open-source licenses, and most of these libraries can be considered de facto standards within their field. Table 6 also illustrates the lightweight nature of NiftyLink and the progressively more heavyweight nature of NiftyIGI.

**Table 5:** The main open-source libraries used in NifTK

Library	License	Purpose	Version
Boost	Boost	General C++	1.56
GDCM	Creatis	DICOM file IO	2.4.1
DCMTK	BSD	DICOM networking	3.6.1
OpenCV	BSD	Computer vision	2.4.6
ITK	Apache 2	Image processing	4.5.1
VTK	BSD	Visualisation	6.1.0
IGSTK	BSD	General IGI functionality, e.g. tracker interfaces	8b8c6f30 (ITK 3.20, VTK 5.8)
OpenIGTLink	BSD	Transmission protocol	972d7b90
CTK	Apache 2	DICOM, infrastructure	1dd16ae7
MITK	BSD like	Application framework	c00657bd
Qt	LGPL	User interface	4.8.6

**Table 6:** Library dependencies for each project in NifTK

Project	Libraries
NiftyLink	Qt, OpenIGTLink
NiftyGuide	Qt, NiftyLink, optionally IGSTK, ITK, VTK and various device API
NiftyIGI	Qt, NiftyLink, ITK, VTK, BOOST, GDCM, DCMTK, CTK, MITK OpenCV

### Software development process

The software build processes are based on the Kitware software process [26], utilising Git^30^ for source code management, CMake^31^ for the cross-platform build process, CTest (see CMake) to run unit tests and subsequently publish continuous and nightly build results to a CDash^32^ dashboard and finally Doxygen^33^ for documentation generation. For the open-source NiftyLink project, all code is available on GitHub,^34^ with issues, bug fixes and feature requests managed using the GitHub issue tracker.

## Footnotes

1
http://slicerigt.github.io/index.html.

2
http://www.commontk.org.

3
http://www.osgi.org.

4
http://cppmicroservices.org.

5
http://www.ndigital.com.

6
http://www.ultrasonix.com.

7
http://qt-project.org.

8
http://github.com/openigtlink/OpenIGTLink.

9
http://www.commontk.org.

10
http://qt-project.org.

11
https://bitbucket.org/razvanpetru/qt-components/wiki/QsLog.

12
https://github.com/openigtlink/OpenIGTLink.

13
https://github.com/NifTK/OpenIGTLink.

14
http://www.cmake.org.

15 Revision 849b434b4b.

16
http://ptpd.sourceforge.net.

17
http://www.nvidia.com.

18
http://www.vikingsystems.com.

19
http://www.ndigital.com.

20
http://www.analogicultrasound.com.

21
http://www.ascension-tech.com.

22
http://www.visiblepatient.com.

23
http://www.vikingsystems.com.

24
http://www.kyotokagaku.com/products/detail03/us-3.html.

25
http://linux.die.net/man/2/send.

26
http://www.meinbergglobal.com/english/sw/ntp.htm.

27
http://cppmicroservices.org.

28
http://github.com/MITK/MITK.

29
http://www.commontk.org.
